# Supplementary material for: Somatic Embryogenesis and Agrobacterium-Mediated Gene Transfer Procedures in Chilean Temperate Japonica Rice Varieties for Precision Breeding
Source: Plants (Basel). 2024 Jan 31;13(3):416. doi: 10.3390/plants13030416 (PMC10857379; doi:10.3390/plants13030416)
Supplement: Supplementary file 1 [file plants-13-00416-s001.zip › Supplementary Tables S1.pdf]

Supplementary Table S1. Culture media for somatic embryogenesis in Chilean temperate japonica rice.

Medium	Composition
Base	$(NH_4)_2SO_4$ 463 mg/L, KNO_3 2.83 g/L, KH_2PO_4 400 mg/L, $MgSO_4$*7H2O 185 mg/L, $CaCl_2$*$2H_2O$ 166 mg/L, H_3BO_3 1.6 mg/L, KI 0.83 mg/L, $MnSO_4$*4H2O 4.4 mg/L, $ZnSO_4$*$7H_2O$ 1.5 mg/L, Na_2EDTA 37.3 mg/L, $FeSO_4$*$7H_2O$ 27.8 mg/L, glycine 2.0 mg/L, thiamine-HCl 1.0 mg/L, pyridoxine-HCl 0.5 mg/L, nicotinic acid 0.5 mg/L, agar 7 g/L
2N6	Base Medium supplemented with myo-inositol 100 mg/L, L-proline 500 mg/L, 2,4-dichlorophenoxyacetic acid (2,4-D) 2.0 mg/L, casein hydrolysate 500 mg/L, sucrose 30 g/L, pH 5.8
N6R	Base medium supplemented myo-inositol 100 mg/L, L-proline 500 mg/L, kinetin 0.5 mg/L, casein hydrolysate 1.0 mg/L, sorbitol 30 g/L, sucrose 20 g/L, activated charcoal 500 mg/L, pH 5.8
N6RN	Base medium supplemented with myo-inositol 100 mg/L, L-proline 500 mg/L, kinetin 0.5 mg/L, 6-bencylaminopurine (BAP) 2.0 mg/L, indole-3-butyric acid (IBA) 1.5 mg/L, 2,4-D 1.5 mg/L, myo-inositol 100 mg/L, L-proline 500 mg/L, casein hydrolysate 1.0 mg/L, sorbitol 30 g/L, sucrose 20 g/L, activated charcoal 500 mg/L, pH 5.8
N6F	Base medium supplemented with casein hydrolysate 1.0 mg/L, sorbitol 30 g/L, sucrose 15 g/L, activated charcoal 500 mg/L, pH 5.8
PIMII	K_2HPO_4 3 g/L, NaH_2PO_4 1 g/L, NaCl 1 g/L, MgSO4*7H2O 0.3 g/L, KCl 0.15 g/L, CaCl2 10 mg/L, FeSO4*7H2O 2.5 mg/L, 2-(N-morpholino) ethanesulfonic acid 75 mM, glucose 1%, pH 5.7
2N6200cc	2N6 supplemented with cefotaxime and carbenicillin 200 mg/L, each (MilliporeSigma)
